# Thermosensitive Poly(DHSe/PEG/PPG Urethane)-Based Hydrogel Extended Remdesivir Application in Ophthalmic Medication

**DOI:** 10.3390/pharmaceutics14010050

**Published:** 2021-12-27

**Authors:** Sennan Xu, Lingjie Ke, Sichen Zhao, Zhiguo Li, Yang Xiao, Yunlong Wu, Jie Ren, Yan Qiu

**Affiliations:** 1 Fujian Provincial Key Laboratory of Ophthalmology and Visual Science, Eye Institute, School of Medicine, Xiamen University, Xiamen 361102, China; 24520191153693@stu.xmu.edu.cn (S.X.); 24520191156040@stu.xmu.edu.cn (S.Z.); 24920172204654@stu.xmu.edu.cn (Y.X.); 2 School of Pharmaceutical Sciences, Xiamen University, Xiamen 361102, China; 32320191153429@stu.xmu.edu.cn (L.K.); lizhiguo@stu.xmu.edu.cn (Z.L.); wuyl@xmu.edu.cn (Y.W.)

**Keywords:** thermosensitive hydrogel, remdesivir, ophthalmic drug delivery, ocular retention, ocular irritation

## Abstract

The spread of infection with severe acute respiratory syndrome coronavirus 2 (SARS-CoV-2) caused the coronavirus disease 2019 (COVID-19) outbreak beginning in March 2020. Currently, there is a lack of suitable dose formulations that interrupt novel coronavirus transmission via corneal and conjunctival routes. In the present study, we developed and evaluated a thermosensitive gelling system based on a selenium-containing polymer for topical ocular continuous drug release. In detail, di-(1-hydroxylundecyl) selenide (DHSe), poly(ethylene glycol) (PEG), and poly(propylene glycol) (PPG) were polymerized to form poly(DHSe/PEG/PPG urethane). The polymer was used to carry poorly water-soluble remdesivir (RDV) at room temperature to form the final thermosensitive in situ gel, which exhibited a typical sol-gel transition at 35 °C. The formed polymer was further characterized by rheology, thermology, and scanning electron microscopy. In vitro release studies and in vivo retention and penetration tests indicated that the thermogel provided the prolonged release of RDV. The RDV-loaded in situ gel was proven to be non-biotoxic against human corneal epithelial cells, with good ocular tolerance and biocompatibility in rabbit eyes.

## 1. Introduction

The ongoing coronavirus disease 2019 (COVID-19) pandemic was caused by the severe acute respiratory syndrome coronavirus 2 (SARS-CoV-2), a *β*-coronavirus within the family Coronaviridae [1]. According to the World Health Organization and Johns Hopkins University dashboard, the global COVID-19 case number stood at approximately 270 million and the death toll was more than 5.3 million by the end of December 2021 (https://www.arcgis.com/apps/dashboards, accessed on 12 December 2021). The nature of the spread of respiratory viruses is typically associated with respiratory droplets, aerosol transmission, and physical contact with respiratory secretions [2,3]. The potential of SARS-CoV-2 to infect the ocular epithelia and mucosa has been highlighted through ocular surface exposure to pollutants and the virus [4]. Considering that the ocular tissues exhibit an anatomical proximity to the respiratory tract, viruses can lead to ocular complications and further cause acute respiratory infection [5,6]. 

Documented reports of the distribution of angiotensin-converting enzyme 2 (ACE2) receptors and transmembrane protease serine 2 (TMPRSS2) protein in ocular tissues brought insights into the potential of SARS-CoV-2 to invade the anterior eye segment [6,7]. The susceptible population may be exposed to COVID-19 by touching the eyes with possibly contaminated hands. The contaminated tears passing down through the nasolacrimal duct to the nasal epithelium also serve as a route for ocular infection. Altogether, these findings shed new light on a possible route for infection by SARS-CoV-2, but this pathway is difficult to protect with ordinary external devices such as masks [8,9].

Remdesivir (RDV) is a broad-spectrum antiviral medication developed by Gilead Sciences (Foster, USA) [10]. The in vitro antiviral effects of RDV are supported by multiple trials [11,12]. On 22 October 2020, the US Food and Drug Administration (FDA) approved Veklury (RDV-loaded cyclodextrin) for the treatment of coronavirus infection. Several formulations (e.g., oral preparation) of RDV, and its parent nucleoside GS-441524, have been projected to expand RDV applications [13]. However, to our knowledge, the application of RDV in ophthalmology has not been reported. The development of RDV eye drops is of benefit to the susceptible population in case of occupational and accidental ocular exposure to SARS-CoV-2.

Ocular application of RDV has proven difficult due to its limited aqueous solubility. It is not possible to enhance the solubility using a heating process because RDV is unstable at high temperatures. Conventional ophthalmic formulations (e.g., eye drops and ointments) suffer from very short retention times at the corneal surface site, which results in poor ocular bioavailability [14]. To provide the possibility to address the abovementioned problems, a thermosensitive hydrogel with great biocompatibility, low toxicity, mucoadhesiveness, and excellent ocular tolerance is proposed in this paper. A thermosensitive triblock copolymer hydrogel containing selenol was developed because selenium acts as an essential trace element to protect the cells of the ocular surface [15,16]. To assess the potential feasibility of the RDV-loaded poly(DHSe/PEG/PPG urethane) thermogel as an ocular delivery system, optical transparency, rheological parameters, thermal properties, and sustained-release behavior were thoroughly characterized. A biocompatibility evaluation in human corneal epithelial cells (HCECs) and in rabbit eyes of the RDV-loaded thermogels was performed to verify the safety of the thermogel. Finally, the thermogel exhibited an increased contact time between the eye surface and enhanced drug permeation into the eye compartments, leading to better control of SARS-CoV-2 infection via the ocular route.

## 2. Materials and Methods

### 2.1. Materials

Di-(1-hydroxylundecyl) selenide (DHSe), polyethylene glycol (PEG), polypropylene glycol (PPG), 1,6-hexamethylene diisocyanate (HMDI), dibutyltin dilaurate (DBT), and paraplast plus were supplied by Sigma-Aldrich (St. Louis, MO, USA). Dulbecco’s Modified Eagle’s Medium/Ham’s F-12 Medium (DMEM/F12) cell culture medium and fetal bovine serum were supplied by Life Technologies (Carlsbad, CA, USA), while 4,6-diamidino-2-phenylindole (DAPI) and phosphate-buffered solution (PBS) were obtained from Beyotime Biotechnology (Shanghai, China). MD25-3.5 dialysis membranes (molecular weight cut-off 3.5 kDa) were purchased from Viskase Companies, Inc. (Chicago, IL, USA). A hematoxylin–eosin (H&E) staining kit was obtained from KeyGEN BioTECH (Nanjing, China) and methanol (HPLC-grade) from Thermofisher (Waltham, MA, USA).

### 2.2. Synthesis of Poly(DHSe/PEG/PPG Urethane)

Poly(DHSe/PEG/PPG urethane) was synthesized by a modified method developed by our group [17]. The detailed synthetic procedure was as follows. Briefly, 10.0 g of PEG, PPG oligomers (PEG/PPG feed at 3/1 for DHSe-31 and PEG/ PPG feed at 1/1 for DHSe-11), and 0.05 g of DHSe-diol were mixed and then dried under vacuum at 40 °C overnight. The moisture content was then removed by azeotropic distillation with 200 mL anhydrous toluene. When the flask was placed in an oil bath at 75 °C under argon, 0.913 g HMDI and two drops of DBT were added. The mixture was further stirred for 48 h. After the preceding steps were completed, the collected copolymer was precipitated in ether and further purified via dialysis using a cut-off membrane. The resultant polyurethane was obtained through a freeze-drying process [18].

### 2.3. Molecular Characterization

Molecular characterization was performed using ^1^H NMR spectra and gel permeation chromatography (GPC). ^1^H NMR spectra were measured in CDCl_3_ solvent using JEOL 500 MHz NMR spectrometers (JEOL USA, Peabody, MA, USA). GPC assay was performed at 40 °C on a Viscotek GPCmax module (Malvern Panalytical, Malvern, UK) equipped with two series-connected Phenogel™ columns (10^3^ and 10^5^ Å, 300 × 7.80 mm). The eluent of GPC was tetrahydrofuran, and the flow rate was set at 1.0 mL/min. GPC profile was acquired with poly(methyl methacrylate), in a Viscotek refractive index detector. 

### 2.4. Determination of Thermal Properties

The thermal properties were determined using differential scanning calorimetry (DSC) and thermogravimetric analysis (TGA). DSC assay was performed using a PDSC-Q100 calorimeter (TA Instruments, New Castle, DE, USA). Samples were heated in a stream of nitrogen, ranging from −80 °C to 120 °C (heating rate of 20 °C per minute). TGA assay was conducted on a TGA Q500 analyzer (TA Instruments, New Castle, USA) under nitrogen at a heating rate of 20 °C per minute from room temperature to 800 °C. Glass transition temperature (*T*_g_) and decomposition temperature (*T*_d_, 5% weight loss) were calculated. 

### 2.5. Determination of Rheological Properties

The rheological properties of the hydrogels were estimated in steady-state and dynamic modes using a DHR-3 rheometer (TA Instruments, New Castle, USA). Oscillatory amplitude sweeps were conducted at a frequency of 1 Hz and the frequency sweeps were conducted at a strain of 1%. All tests were performed at 37.0 °C. Storage modulus (*G′*) and loss modulus (*G″*) were measured as functional parameters.

### 2.6. Determination of Sol-Gel Phase Transition

Sol-gel phase transition was determined using an inverting vial method and a stirrer method in increments of 2 °C per minute [19,20]. A series of different concentrations (2 to 20 *w*/*v*%) of poly(DHSe/PEG/PPG urethane) copolymer systems were prepared. The inverting vial test consisted of equilibration at 4 °C, followed by immersion for 5 min in a water bath. Gelation temperature (GT) was defined if the gel was firm enough over 1 min after inversion. As for the stirrer method, a magnetic bar and 5 mL of sample solution were placed into a 25 mL transparent vial. A mercury thermometer was kept perpendicularly positioned 3 mm from the bottom. The solution was heated with continuous stirring of 100 rounds per minute. The temperature at which the magnetic bar stopped turning was recorded as GT. In addition, the minimum gelation concentration (MGC) was defined as the minimum copolymer concentration at which the gelation behavior could be observed.

### 2.7. Cryo-Scanning Electron Microscopy

The microscopic appearance of the hydrogel was observed using a Quanta™ 450 FEG high-resolution SEM (FEI Company, Hillsboro, OR, USA), which was equipped with a PP3000T preparation and a cryo-transfer system (Quorumtech, Lewes, UK). During the cryo-scanning electron microscopy (Cryo-SEM) procedure, a small piece of the hydrogel sample (ca 2 mm × 2 mm × 2 mm) was immersed in liquid nitrogen for 30 s and then transferred to the cryogenic reparation chamber using the cryo-preparation and transfer system. The frozen specimens were cut into thin sections for SEM observation, followed by automatic sublimation (−90 °C, 10 min) and sputtering (10 mA, 60 s). Hydrogel morphology was observed in low-vacuum mode at an accelerating voltage of 5 kV; the cooling stage was set at −140 °C.

### 2.8. Preparation of RDV-Loaded Poly(DHSe/PEG/PPG Urethane) Hydrogel

Synthesis of RDV-loaded poly(DHSe/PEG/PPG urethane), namely RDV/poly(DHSe/PEG/PPG urethane), was performed using a two-step method at 4 °C. Five or 10 mg RDV and 10 mg poly(DHSe/PEG/PPG urethane) were weighed into 300 μL tetrahydrofuran (THF) to form final 1 mg/mL and 2 mg/mL RDV-loaded hydrogels. The mixture was then added into 700 μL deinonized water using a slow drip with sonication. THF was then volatilized overnight, and equal volume deionized water was readded to form RDV-loaded micelles (Reagent A). Meanwhile, 800 μL aqueous solution of 3 *w*/*v*% poly(DHSe/PEG/PPG urethane) was equilibrated at 4 °C to obtain Reagent B. Finally, 200 μL reagent A was mixed with reagent B with stirring and sonication at 4 °C.

### 2.9. Determination of Light Transmittance

The optical transmittance of the hydrogel was estimated by comparing the RDV/poly(DHSe/PEG/PPG urethane) with RDV-loaded poloxamer (brand name Pluronic) hydrogel at 4 °C and 37 °C. The samples were first observed after being dripped onto a letter “A” card. Photographs were obtained using an Eclipse E400 camera (Nikon, Kyoto, Japan). The absorbance of all samples was then determined by continuous scanning within 380–780 nm range using an ultraviolet spectrophotometer (UV-2600, Shimadzu, Kyoto, Japan) and the results were combed with Shimadzu UVprobe2.42 software.

### 2.10. In Vitro Drug Release Study

RDV-loaded cyclodextrin inclusion complex was prepared as a control before the drug release study. Literature methods were followed as references [21,22]. The in vitro release profiles of RDV from the thermogels and cyclodextrin inclusion complexes were measured in Meilubio artificial tears (Dalian, China) at 35 °C. Briefly, 200 μL of RDV-loaded thermogel and cyclodextrin inclusion compound (both 1 mg/mL) were sealed into an MD25-3.5 dialysis membrane. Then, 2 mL of artificial tears was used as release medium. At predetermined time points, 1 mL of release medium was extracted, and 1 mL of artificial tears was replenished to maintain the volume. Cumulative release (%) profile of RDV was calculated using the Agilent 1200 HPLC system (Agilent Poroshell 120 EC-C18, 4.6 × 150 mm i.d., 4 µm) with a mobile phase of 0.05% TFA in H_2_O/acetonitrile (50:50 *v*/*v*) at 254 nm. The column temperature was 30 °C and the injection volume was set at 10 µL. The total run time was 31.0 min with a flow rate of 1.0 mL/min; the peak at around 18.5 min was assigned to RDV.

### 2.11. Determination of Precorneal Fluorescent Dye Retention

Fluorescein sodium cyclodextrin solution and poly(DHSe/PEG/PPG urethane) containing fluorescein sodium were used to determine the fluorescent dye retention. Briefly, 20 μL fluorescein-containing eye drops was topically instilled twice to the ocular surface of rabbit eyes. The rabbits could blink as normal. Subsequently, the cornea and conjunctiva were imaged at predetermined points (1, 15, 30, and 60 min) by an experienced oculist with a slit-lamp microscope with blue fluorescence (Leica, Wetzlar, Germany). The animal experiment in rabbits was approved in June 2019 by the Experimental Animal Ethics Committee of Xiamen University (IACUC: xmulac20190063).

### 2.12. Imaging of Optical Coherence Tomography

Corneal retention after topical administration of various formulations was determined using optical coherence tomography (OCT). C57BL/6 mice (female, 6–8 weeks of age) were anesthetized using 1% Sumianxin-II (Shengda, Dunhua, China). OCT images were captured using a telecentric lens and an Optoprobe isOCT system (Optoprobe Science, Burnaby, BC, Canada). All images were obtained using volume scanning mode (volume size: width = 2048, height = 600, frames = 600; data process: C2 = −43, Fcoeff = 0.3, max Val = 40, min Val = 20; XR-YR = 2500 mV × 2500 mV; d1/d2/d3 = 100/10/5). After topical administration of 10 μL of the eye drops and manual eyelid blinking, OCT images were obtained at predetermined points.

### 2.13. Measurement of RDV Penetration in Ocular Tissues

After 20 μL of RDV-containing drops was administered twice to the ocular surface in 1 min, the rabbits were euthanized and corneas, conjunctiva, and aqueous fluid were collected [23]. All harvested tissues were weighed and stored at −80 °C for 1 h before ultra-performance liquid chromatography combined with tandem mass spectrometry (UPLC-MS/MS) analysis [24]. 

Before extraction, the samples were homogenized in 500 μL distilled deionized water using a KZ-III-F Tissue Lyser (Servicebio, Wuhan, China). RDV was extracted from the homogenates using 1 mL of cold methanol containing 100 ng/mL of the internal standard substance (ISS). The ISS SML-2875, a nucleic acid analogue, was a generous gift from Prof. Zheng Xiao (Xiamen University, Xiamen, China). The solution was then centrifuged at 12,000 rpm and the supernatant was dried down using nitrogen air. The precipitates were then reconstituted in a 150 μL mobile phase and transferred to an autosampler vial. RDV was measured using UPLC-MS/MS, and its concentrations in ocular samples were calculated.

A Waters Acquity I-class UPLC system (Waters, Milford, MA, USA) combined with AB SCIEX QTRAP 6500 mass spectrometric (AB Sciex, Framingham, MA, USA) was used for the analysis. Chromatograms were obtained with an Acquity UPLC HSS T3 column (Waters, 2.1 × 100 mm, 1.8 μm) using a gradient (Table S2). Positive electrospray ionization was used for all analyses. The column temperature was set at 40 °C. The total run time was 6.00 min with a flow rate of 0.4 mL/min. Multiple reaction monitoring traces were quantified as: 603.2 > 402.2 for RDV and 330.1 > 128.0 for ISS. All parameter settings are detailed in Tables S3 and S4. Standard calibration curves were constructed using five concentrations (1.6, 8, 40, 200, and 1000 ng/mL).

### 2.14. Cell Culture

HCECs were obtained from the Eye Bank of Affiliated Xiamen Eye Center of Xiamen University (Xiamen, Fujian, China). HCECs were cultured in 6% fetal bovine–DMEM/F12 medium (Life Technologies, Waltham, MA, USA), containing 10 ng/mL human epidermal growth factor (Peprotech, East Windsor, NJ, USA), 10 μg/mL insulin (Sigma-Aldrich, USA), and 100 units/mL penicillin/streptomycin antibiotics (HyClone, Logan, UT, USA). Cells were then seeded on plastic dishes and incubated in a Galaxy 170S CO_2_ incubator (Eppendorf, Hamburg, Germany).

### 2.15. Evaluation of Cytotoxicity

To evaluate cytotoxicity, RDV hydrogels were extracted using 5 mL of DMEM/F12 medium to obtain a standard leach liquor [25]. The HCECs were seeded onto a 96-well plate with 1 × 10^4^ cells per well and then co-cultured with 100 μL leaching liquor. The morphological appearance of HCECs was observed using a DM IL LED microscopy (Leica, Germany) to obtain a sense of immediacy. Cell proliferation capacity was measured following the instructions of the Cell Counting Kit-8 (https://www.dojindo.eu.com/TechnicalManual/Manual_CK04.pdf, accessed on 12 December 2021, CCK-8, Dojindo, Japan). 

The rate of apoptosis in HCECs was measured in all groups using the TUNEL assay. This assay identified DNA breaks by labeling the 3′-OH terminals of DNA with modified nucleotides to TdT or terminal nucleotidyltransferase. Briefly, HCECs were cultured on slides and then treated with the leach liquor. After an incubation period of 48 h, the existence of apoptotic cells in the samples was investigated using a DeadEnd™ Fluorometric TUNEL System (Promega, Madison, WI, USA) in accordance with the protocol of the manufacturer. 

### 2.16. Evaluation of Rabbit Eye Irritation

First, 100 μL hydrogels of 1 mg/mL and 2 mg/mL were administrated topically to the left eyes of rabbits, while the right eyes served as the negative control. The left eyes were then observed with a SL9900 Digital LED slit lamp (CSO Elite, Toronto, ON, Canada) during the entire study period. To assess the adverse effects, intra-ocular pressure (IOP) was obtained using an iCare TV02 tonometer (iCare Finland Oy, Vantaa, Finland) between 5 min and 2 h after topical hydrogel administration. IOP was monitored five times in the left eyes of each rabbit and the median was selected for statistical analysis. 

New Zealand rabbits (male, aged 3–4 months) were provided by the Experimental Animal Center of Xiamen University. The animal experiment in rabbits complied with the Association for Research in Vision and Ophthalmology Statement for the Use of Animals in Ophthalmic and Vision Research and was approved by the Experimental Animal Ethics Committee of Xiamen University (IACUC: xmulac20190063). 

### 2.17. Histological Analysis

After the irritation test was completed, H&E staining was performed to investigate the safety of the gelling in vivo. Corneas were carefully dissected and washed twice with physiological saline. The samples were then fixed in 4% formaldehyde. The fixed samples were dehydrated using different gradient concentrations of alcohol. Then, ocular tissues were embedded in paraplast plus and cut into 5-μm-thick sections. Histological sections were fixed in 4% paraformaldehyde, and then stained using the KeyGEN H&E staining kit according to the kit instructions. 

### 2.18. Assay of Surface Plasmon Resonance

Surface plasmon resonance (SPR) assays were performed using a Biacore T200 system and an SPR Chip CM5 of GE Healthcare (Marlborough, MA, USA) as the manual of protocols. Samples, over a range of 0.2–5.0 μmol/L, were injected with PBS containing 5% DMSO (*v*/*v*) at a flow rate of 30 μL/min. Each injection was done with 120 s association and dissociation time. Then, the binding activity of the hydrogel and vehicle to the spike protein of SARS-CoV-2 was investigated. *K*_D_ values were calculated using a steady affinity state model with the BIAcore T200 analysis software of GE Healthcare. 

## 3. Results and Discussion

### 3.1. Synthesis and Characterization of Poly(DHSe/PEG/PPG Urethane)

**Figure 1:**
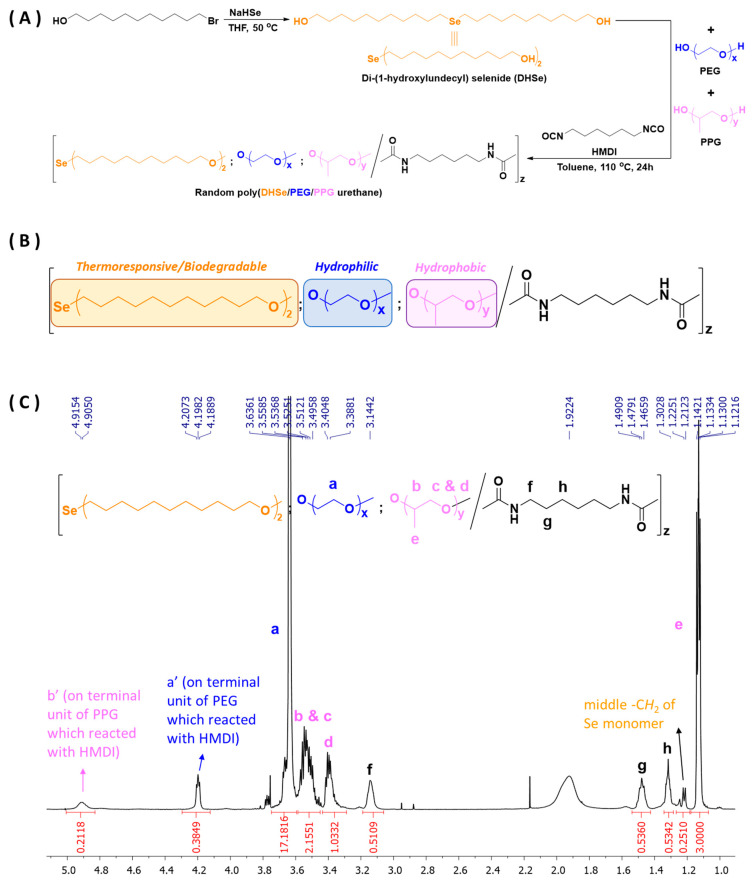
Synthesis and characterization of poly(DHSe/PEG/PPG urethane): (**A**) synthetic scheme; (**B**,**C**) structure and ^1^H NMR spectra of copolymer.

Multi-block copolymers of poly(DHSe/PEG/PPG urethane) were successfully synthesized by the condensation of selenide-containing diols with PEG and PPG oligomers under modified conditions and were characterized using ^1^H NMR (Figure 1). The synthesis process (Figure 1A) was carried out under modified conditions, with excess HDMI as the coupling reagent and dibutyltin dilaurate as the catalyzer. The poly(DHSe/PEG/PPG urethane) structure was determined by ^1^H NMR spectroscopy (Figure 1C). For a more detailed description, the CH_2_ component peak of PEG was located at 3.64 ppm, and proton signals of PPG were located at 3.56–3.39 ppm, while overlapped peaks at 1.14–1.22 ppm were assigned to middle -CH_2_ of Se monomer. The CDCl_3_ proton peak (7.26 ppm) was used as a reference. The molecular weights and dispersities (*Đ*_M_ = *M*_w_/*M*_n_) of the copolymers are tabulated in Table 1. 

### 3.2. Sol-Gel Phase Transition

**Figure 2:**
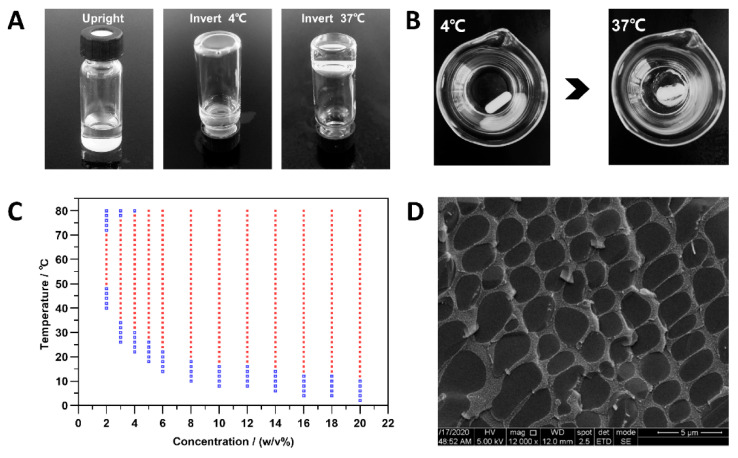
Sol-gel transition was tested by an inverting vial method (**A**) and a stirrer method (**B**); (**C**) phase diagrams of 2–20 *w*/*v*% copolymer aqueous solution (“■” gelation occurred, “🞑” solution condition); (**D**) Cryo-SEM image of the copolymer.

Sol-gel phase transition behavior was inspected using an inverting vial method (Figure 2A) and a stirrer method (Figure 2B). From Figure 2C, gelation occurred for the copolymer when polymer concentrations increased to a specific range; the GT decreased with copolymer concentration increase. The poly(DHSe/PEG/PPG urethane) performed reversible sol-gel transition within a 2–20 *w*/*v*% concentration range. Furthermore, the gels were stable up to a higher temperature. An important advantage of poly(DHSe/PEG/PPG urethane) is the ease of optimizing the GT for different applications by altering its concentration flexibly. The MGC of poly(DHSe/PEG/PPG urethane) copolymers was approximately 3 *w*/*v*% to accommodate to ophthalmic drug delivery. At 3 *w*/*v*% concentration, the triblock copolymer can form a solution state at room temperature and gel at the temperature of the ocular surface, which is favorable for the topical ocular delivery of RDV. The thermogels described in the remaining part of this paper are the 3 *w*/*v*% concentration poly(DHSe/PEG/PPG urethane).

Additionally, polymerized materials with high moisture content were prone to lose water in vacuum, rendering the structural characterization difficult to perform [26]. In this paper, Cryo-SEM with technical assistance by a cooling stage and a liquid nitrogen pool (quick freeze so ice crystals did not have as much time to form) was applied to confirm the morphological results for the hydrogels. The porous structure of the hydrogel was confirmed with a pore size range of 2 to 10 μm (Figure 2D).

### 3.3. Thermal and Rheological Properties

**Figure 3:**
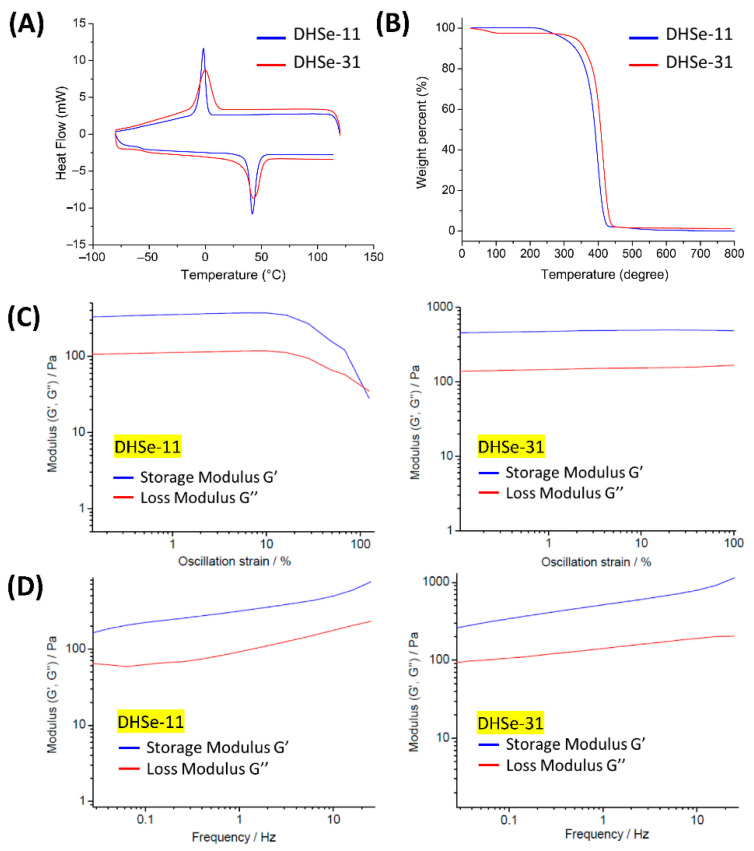
Thermal and rheological properties of hydrogels: (**A**,**B**) DSC and TGA results; (**C**) oscillatory strain sweep plots; (**D**) frequency sweep of DHSe-11 and DHSe-31 hydrogel.

The copolymers exhibited a favorable thermal characteristic. Only one *T*_g_ value was observed, indicating DHSe/PEG/PPG miscibility (Figure 3A,B and Table 1). TGA and DSC studies showed that DHSe-11 and DHSe-31 hydrogels were thermally stable. The poly(DHSe/PEG/PPG urethane) was further characterized by rheological methods. The oscillation dependence of the gels was tested; DHSe-11 hydrogel breaks under high strain, while the DHSe-31 hydrogel remained stable in the whole range of the tested strain (Figure 3C). In the frequency dependence test (Figure 3D), mechanical properties increased linearly with frequency. The thermogel was stable throughout the experiment, with G′ > G″. Both hydrogels are stable in the tested range of frequency. According to the above results, the DHSe-31 hydrogel showed a higher modulus in both the amplitude and frequency sweep test; thus, the feed ratio of PEG and PPG oligomers was set at 3:1. 

### 3.4. Transparency of RDV/Poly(DHSe/PEG/PPG Urethane) Hydrogel

**Figure 4:**
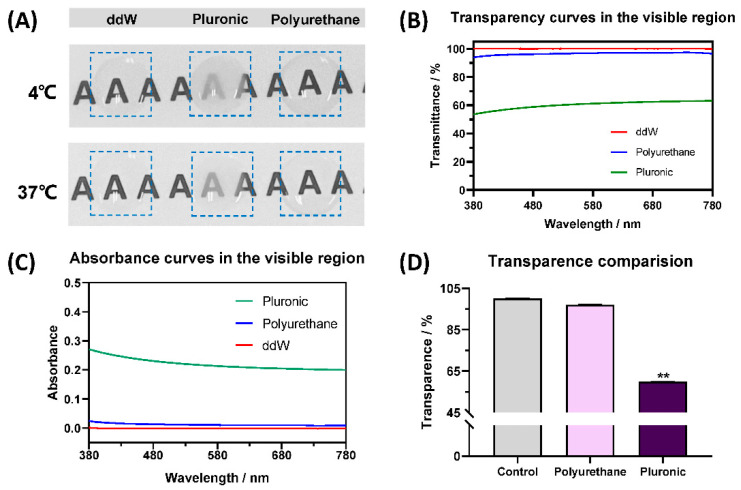
Optical properties of hydrogels: (**A**) macroscopic evaluation of transmittance performance; (**B**,**C**) transmittance and absorbance curves versus visible wavelength; (**D**) transmittance comparison (** *p* < 0.01).

As an important property of ophthalmic biomaterials, biomedical materials exhibiting transparency of over 90% versus visible wavelengths are considered to be highly transmissive. Through the macroscopic examination of a letter “A” card, the clarity of the RDV/poly(DH-Se/PEG/PPG urethane) hydrogel was macroscopically observed (Figure 4A). The RDV/poly(DHSe/PEG/PPG urethane) hydrogel showed clearer visibility when compared with the RDV-loaded poloxamer hydrogel. Absorbance curves in the visible region were also analyzed to perform a thorough and quantitative evaluation (Figure 4B). Transmittance curves at different wavelengths were further calculated (Table S1, Figure 4C,D). It can be clearly seen that the poloxamer hydrogel had poorer transmittance performance compared with RDV/poly(DHSe/PEG/PPG urethane).

### 3.5. In Vitro RDV Release Behavior

**Figure 5:**
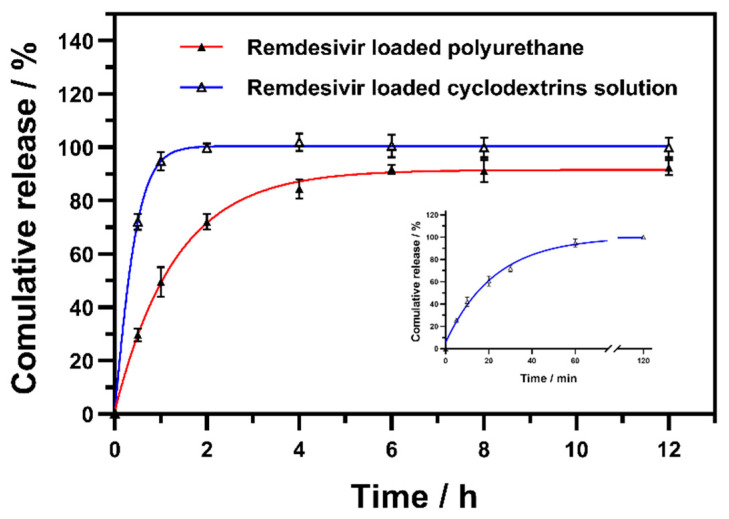
In vitro RDV release profiles of thermogel and cyclodextrin inclusion complex in artificial tears.

To further demonstrate the feasibility of the polyurethane-based thermogel as an RDV carrier, a drug release study was performed. The release profile showed that the cyclodextrin inclusion system exhibited the rapid release of RDV within 1 h (Figure 5). Conversely, it could be seen that the thermogel provided the sustained/prolonged release of 80% RDV in 4 h. The slow releasing of RDV might be attributed to the presence of a reticular structure inside the poly(DHSe/PEG/PPG urethane) hydrogel. Moreover, the in vitro RDV release behavior conformed to the diffusion-based dissolution model [18,20].

### 3.6. Drug Retention and Penetration Test

**Figure 6:**
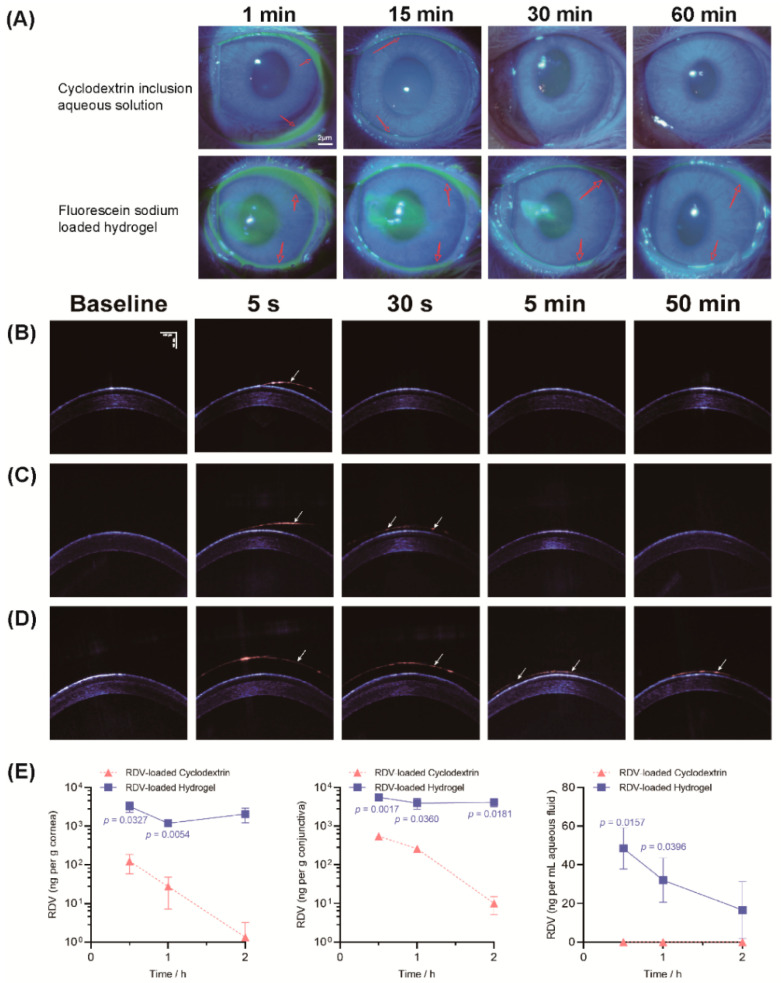
Precorneal retention and RDV penetration results: (**A**) fluorescent dye test; (**B**) PBS; (**C**) cyclodextrin inclusion compound; (**D**) hydrogel retention behavior using OCT imaging; (**E**) RDV concentration in ocular tissues after instillation.

The retention time is an important characteristic of eye drops. As shown in Figure 6A, the fluorescein sodium retention was extended to around 60 min by introducing poly(DHSe/PEG/PPG urethane). Given the universal quenching rate of the fluorescent probe, the accurate prolonged retention time might be relatively longer than 60 min. Conversely, the fluorescein sodium clearance was rapid in the cyclodextrin inclusion compound, and fluorescence staining was almost invisible at 15 min. Compared with a chitosan-based hydrogel [21], poly(DHSe/PEG/PPG urethane) provided a relatively longer-term precorneal retention time (over 60 min vs. 30 min), thus effectively resisting the effect of tear drainage.

OCT was applied to visualize RDV retention behavior. PBS was used to simulate the precorneal retention behavior of commonly used clinical eye drops. A hydration film, structured in close proximity to the corneal surface, was formed immediately after topical PBS administration and was readily cleared due to blinking of the eyelids (Figure 6B). The cyclodextrin inclusion complex aqueous solution, which should behave as a viscous liquid, exhibited an uneven hydration film as with PBS, and was sustained for a short time (Figure 6C). In contrast, rapid gelation of the poly(DHSe/PEG/PPG urethane) was observed and resulted in a thick, well-distributed coating on the cornea (Figure 6D). The gelation layer remained on the ocular surface after 50 min. This result was in agreement with the result of the fluorescein sodium retention test.

UPLC-MS/MS was used to calculate the concentration of RDV penetration in ocular tissues. The retention times were 3.31 min for RDV and 2.59 min for ISS (Figure S1). The linearity range for RDV was from 1.6 ng/mL to 1000 ng/mL, while the limit of detection was 0.97 ng/mL (Figure S1). RDV in the poly(DHSe/PEG/PPG urethane) hydrogel provided significantly higher drug concentrations in rabbit corneas (0.5 and 1.0 h) compared with the cyclodextrin inclusion compound (Figure 6E). We found a similar trend in the conjunctiva, and the greater concentrations of RDV were significant at all points. The RDV in the cyclodextrin inclusion compound solution was indetectable in the aqueous fluid. Despite the low concentration (several tens of nanograms), we found a consistent increase in the RDV/poly(DHSe/PEG/PPG urethane) hydrogel formulation. We found that poly(DHSe/PEG/PPG urethane) effectively enhanced RDV permeation into the anterior ganglion tissue more than that of cyclodextrin. The prolonged RDV retention on the ocular surface is a possible reason for this result.

### 3.7. In Vitro Cytotoxicity 

**Figure 7:**
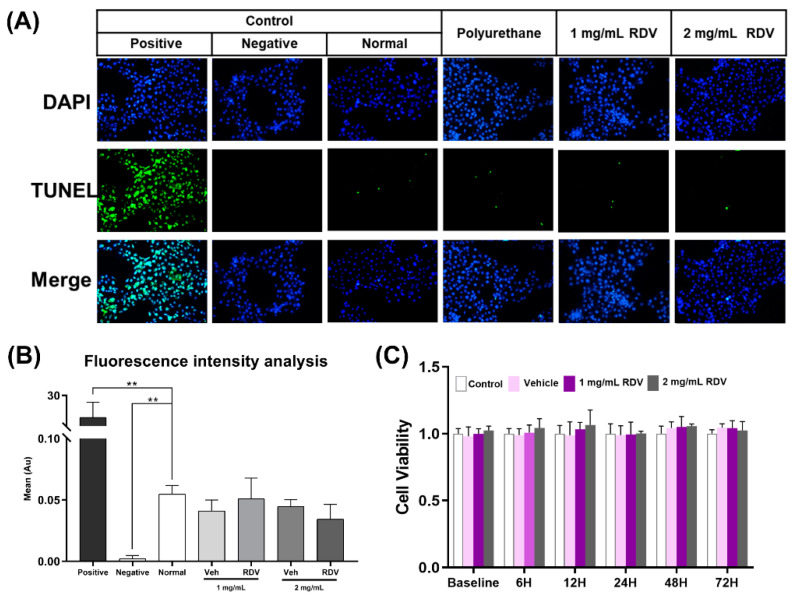
In vitro cytotoxicity result: (**A**) TUNEL staining of HCECs after different treatments; (**B**) fluorescence intensity analysis of TUNEL assay (mean ± SD, ** *p* < 0.01 vs. normal); (**C**) proliferation ability assay by CCK-8.

In general, the cell morphology results showed that the structure and density of HCECs treated with leach liquor were normal (Figure S2). Results of the TUNEL assay and fluorescence intensity analysis are shown in Figure 7A,B. There was a significant difference between the normal group and the positive control (treated with DNase I) or the negative control, in terms of apoptosis measured with TUNEL staining (*p* < 0.01). Compared with the normal group, no significant differences were observed after treatment with leach liquor, according to the fluorescence intensity Arbitrary Unit (A.U.) analysis. As shown in Figure 7C, the results of the CCK-8 assay on HCECs indicated that RDV/poly(DHSe/PEG/PPG urethane caused minimal cytotoxicity (cell viability > 90%) for HCECs after 72 h of incubation. This result suggests that the thermogel system might act as a safe vehicle for ophthalmic drug delivery. 

### 3.8. Ocular Irritation and Histological Evaluation

**Figure 8:**
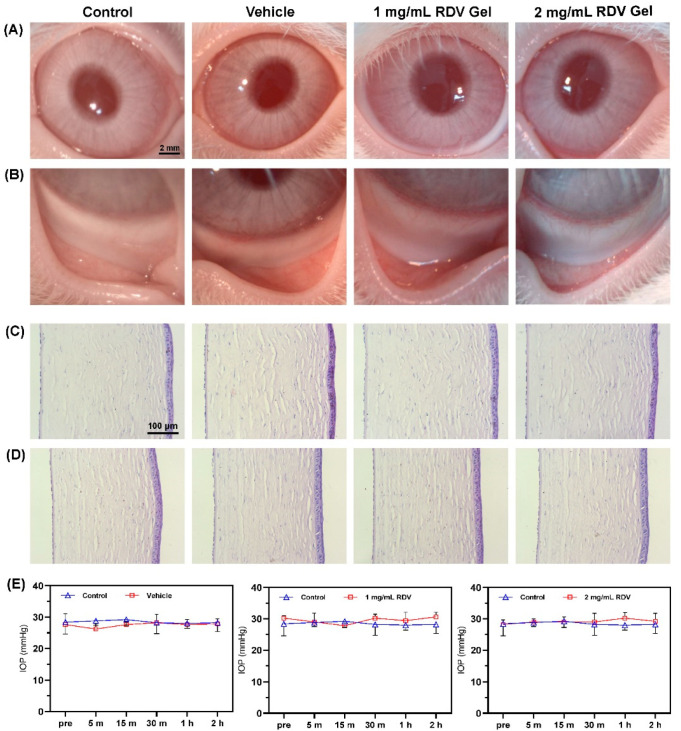
Ocular irritation observation and histological evaluation: (**A**,**B**) corneal and conjunctival toleration under the slit-lamp microscope; (**C**,**D**) H&E staining of center and peripheral region of cornea; (**E**) IOP of rabbits after treatment.

After a single topical administration of the RDV/poly(DHSe/PEG/PPG urethane) hydrogel and its carrier, no signs of conjunctival congestion and edema, corneal epithelial exfoliation, or opacification appeared in all groups, suggesting that the hydrogels were well-tolerated (Figure 8A,B). Post-drop application, IOP in rabbits treated with hydrogels remained stable during the test period (one-way ANOVA test, control group vs. 1 mg/mL RDV hydrogel group (*p* = 0.0516), and control group vs. 2 mg/mL RDV hydrogel group (*p* = 0.3307)). In the eyes treated with the carrier, the mean IOP varied widely, with a low average, but no significant difference was observed (Figure 8E). The results of IOP demonstrated that poly(DHSe/PEG/PPG urethane) showed no IOP-lowering effect after administration for 2 h, and the change in IOP was also more stable than poloxamer, carbopol, and PU/HPCD [20].

After the irritation test, histopathological examination of the corneas was used to evaluate the hydrogels’ safety. The top row of the figure illustrates the central cornea (Figure 8C), and the following row illustrates the peripheral region of the cornea (Figure 8D). After ocular administration, corneal cells possessed a clear outline and were tightly arranged, without epithelial defects and inflammatory cell infiltration. H&E sections of corneas revealed normal epithelium linings and corneal stroma, including the corneal tissues of rabbits that received the hydrogel vehicle and RDV-containing hydrogel. Thus, the hydrogel was well-biocompatible for rabbit eyes.

### 3.9. SPR Analysis with Spike Glycoprotein

**Figure 9:**
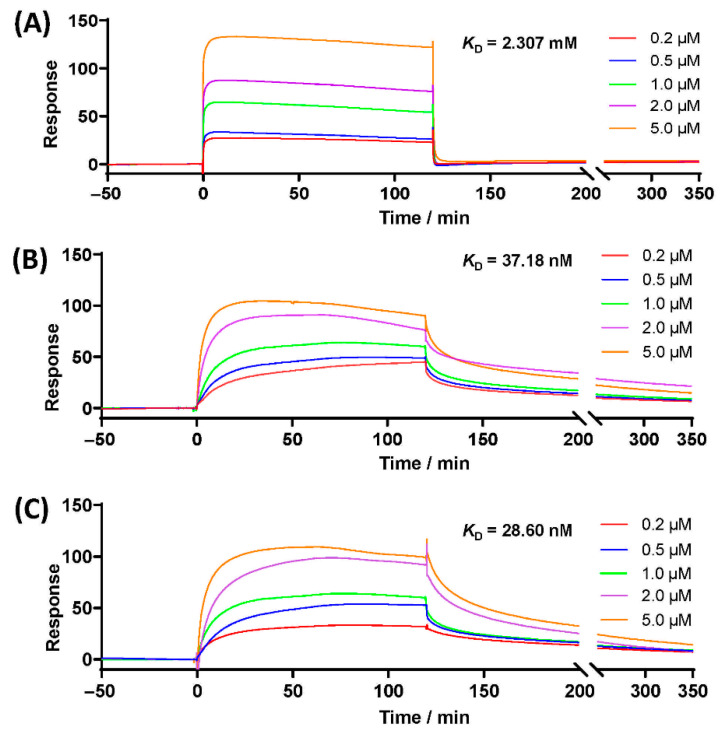
SPR biosensor analysis: (**A**) poor affinity between vehicle and spike protein (*K*_D_ = 2.307 mM); (**B**) favorable binding of RDV and spike protein (*K*_D_ = 37.18 nM); (**C**) the binding would not be blocked by hydrogel carrier (*K*_D_ = 28.60 nM).

The binding affinities of RDV and spike glycoprotein were verified in vitro. As shown in Figure 9, the biosensor responses to the vehicle were significantly low due to the nonspecific interaction with the spike protein (*K*_D_ = 2.307 mM, Figure 9A). However, the interactions between the hydrogel and the SARS-CoV-2 spike protein were concentration-dependent. The *K*_D_ value between RDV and spike glycoprotein was 28.60 nmol/L (Figure 9B), indicating a strong affinity. The response curve maintained a good affinity (*K*_D_ = 37.18 nM) after RDV with loaded with polyurethane (Figure 9C). These results suggest that using an RDV hydrogel is an efficient strategy for the prevention of SARS-CoV-2 infection, given that affinity was not blocked by the hydrogel carrier. Meanwhile, this result indicates a potential pathway of RDV preventing SARS-CoV-2 transmission, not only through inhibiting RNA polymerase. Follow-up studies are required to verify this result.

## 4. Conclusions

In this work, an RDV-loaded poly (DHSe/PEG/PPG urethane) thermogel was successfully developed as a topical ocular delivery system. Compared with the previously reported triblock copolymer, the proposed poly(DHSe/PEG/PPG urethane) hydrogel formed was prepared at different feed ratios of PEG and PPG. The RDV-loaded polyurethane hydrogel was prepared using a two-step method in a low-temperature environment. The polyurethane thermogel had a typical sol-gel transition at 35 °C and showed favorable optical, rheological, and thermal properties. More importantly, the RDV/poly(DHSe/PEG/PPG urethane) hydrogel system at an eye drop dose caused minimal toxicity against HCECs and rabbit eyes. To the best of our knowledge, this is the first attempt to develop a drug delivery system using RDV loaded into a hydrogel. Compared with aqueous solution formulation, the RDV/poly(DHSe/PEG/PPG urethane) hydrogel significantly enhanced the ocular retention and penetration, thus providing a potential method to block SARS-CoV-2 transmission via the ocular surface.

## Figures and Tables

**Table 1:** Molecular characteristics and thermal properties of poly(DHSe/PEG/PPG urethane).

Sample	Feed Ratio		Thermal Analysis			GPC	
	PEG	PPG	*T*_m_*^a^*/°C	*T*_g_*^a^*/°C	*T*_d_*^b^*/°C	*M*_n_/Da	*Đ*
DHSe-11	1	1	41.85	−57.11	298	32,018	1.14
DHSe-31	3	1	43.26	−55.77	332	25,523	1.09

*^a^ T*_m_ and *T*_g_ values obtained from DSC analysis (photo differential scanning calorimeter equipped with an auto cool accessory and calibrated using indium, −80 °C to 120 °C, 20 °C/min); *^b^ T*_d_ value obtained from TGA analysis with a heating rate of 20 °C/min from room temperature to 800 °C.

## Data Availability

The data presented in this study are available on request from the corresponding author.

## Supplementary Materials

The following are available online at https://www.mdpi.com/article/10.3390/pharmaceutics14010050/s1, Figure S1: (A) The retention times for RDV and ISS; (B) linearity range of RDV, Figure S2: Morphological appearance of HCECs at different magnifications, Table S1: The transparence of three systems at different wavelengths (%), Table S2: Chromatographic gradient, Table S3: General instrument settings, Table S4: Analyte-specific parameters.
