# Supplementary material for: Thermosensitive Poly(DHSe/PEG/PPG Urethane)-Based Hydrogel Extended Remdesivir Application in Ophthalmic Medication
Source: Pharmaceutics. 2021 Dec 27;14(1):50. doi: 10.3390/pharmaceutics14010050 (PMC8778792; doi:10.3390/pharmaceutics14010050)
Supplement: Supplementary file 1 [file pharmaceutics-14-00050-s001.zip › pharmaceutics-1488753-supplementary.pdf]

pharmaceutics

Supplementary Material: Thermosensitive Poly(DHSe/PEG/PPG Urethane)-Based Hydrogel Extended Remdesivir Application in Ophthalmic Medication

Sennan Xu, Lingjie Ke, Sichen Zhao, Zhiguo Li, Yang Xiao, Yunlong Wu, Jie Ren and Yan Qiu

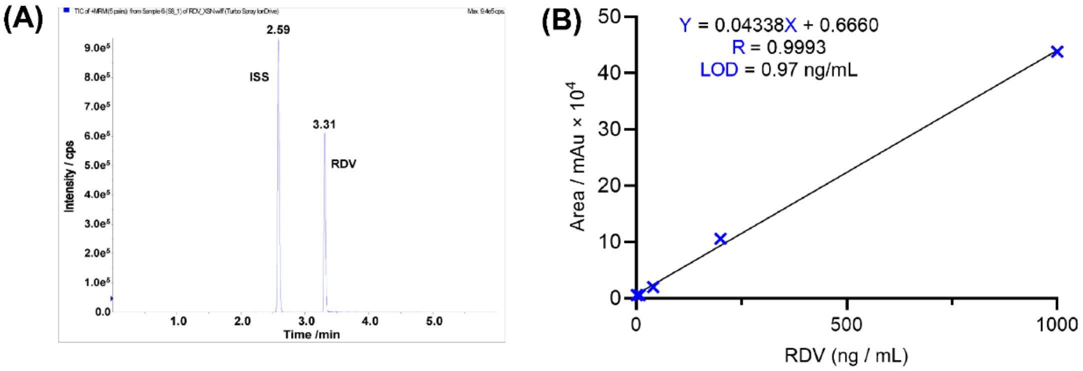

Figure S1. (**A**) The retention times for RDV and ISS; (**B**) linearity range of RDV.

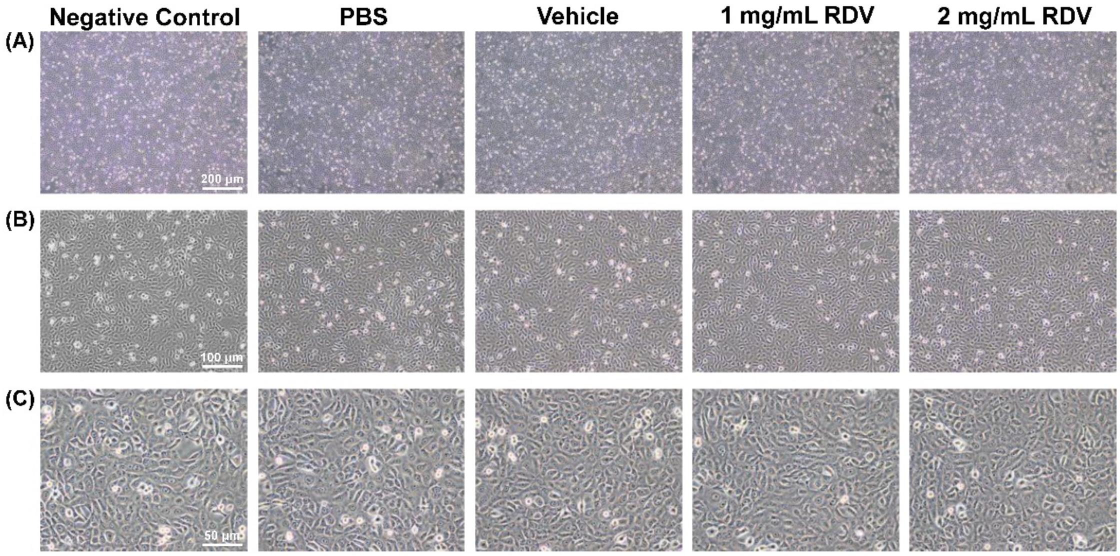

Figure S2. Morphological appearance of HCECs at different magnifications.

Table S1. The transparence of three systems at different wavelength (%).

Group	Wavelength							Mean
	420	450	485	550	590	600	700	
ddW	100.1 ± 0.1	100.2 ± 0.1	100.1 ± 0.1	100.2 ± 0.1	100.2 ± 0.1	100.2 ± 0.1	100.2 ± 0.0	100.2 ± 0.1
Polyurethane	95.79 ± 0.3	96.24 ± 0.3	96.60 ± 0.3	97.20 ± 0.3	97.57 ± 0.4	97.57 ± 0.4	97.80 ± 0.6	96.97 ± 0.3
Pluronic	56.12 ± 0.1	57.60 ± 0.1	58.93 ± 0.1	60.64 ± 0.4	61.36 ± 0.3	61.51 ± 0.4	62.62 ± 0.0	59.83 ± 0.6

Table S2. Chromatographic gradient.

Time (min)	% Phase A	% Phase B
0.00	95	5
0.50	95	5

2.00	70	30
2.50	30	70
3.50	5	95
4.00	5	95
4.10	95	5
6.00	95	5

Phase A: 0.1% Formate in water; Phase B: 0.1% Formate in acetonitrile.

Table S3. General instrument settings.

Variables	Setting
Ion mode	ESI+
Ion source	Turbo Spray IonDrive
Curtain Gas	35 psi
Collision Gas	medium
IonSpray Voltage	5500 V
Source temperature	500 ℃
Ion Source Gas 1	55 psi
Ion Source Gas 2	60 psi

Table S4. Analyte-specific parameters.

Variables	Parent Ion (m/z)	Product Ion (m/z)	Declustering Potential (V)	Collision Energy (V)
RDV	603.2	402.2	40	22
	603.2	318.1	40	30
ISS	330.1	128.0	80	23
	330.1	203.1	80	14
